# Pancreatitis: correcting CFTR expression and function as a promising effective treatment

**DOI:** 10.3389/fphys.2026.1813824

**Published:** 2026-05-01

**Authors:** Paramita Sarkar, Wei-Yin Lin, Ava Movahed Abtahi, Woo Young Chung, Shmuel Muallem

**Affiliations:** The Epithelial Signaling and Transport Section, National Institute of Dental and Craniofacial Research, National Institutes of Health, Bethesda, MA, United States

**Keywords:** CFTR, correctors, pancreatitis, potentiators, treatment

## Abstract

The two principal cell types of the exocrine pancreas, acinar and ductal cells, serve distinct but complementary roles. Acinar cells synthesize and secrete digestive enzymes, whereas duct cells secrete fluid and bicarbonate (HCO_3_^-^). Ductal secretion protects the pancreas by producing an alkaline fluid that prevents premature digestive enzyme activation and facilitates their transport to the intestine. A critical step in this protective mechanism is protein kinase A–mediated activation of the luminal Cl^-^ channel, cystic fibrosis transmembrane conductance regulator (CFTR). In this context, increasing evidence indicates that impaired CFTR function represents a common pathological feature across all forms of pancreatitis, the most prevalent disease of the exocrine pancreas. Acute and chronic pancreatitis are inflammatory disorders characterized by pancreatic ductal injury that initiates recurrent inflammatory episodes and subsequent acinar cell damage, for which effective treatments remain lacking. Accordingly, this review aims to summarize current evidence supporting alterations in CFTR expression and activity as key events in pancreatitis pathogenesis. Furthermore, given the availability of safe and effective CFTR correctors and potentiators currently approved for the treatment of cystic fibrosis, we argue that these agents warrant strong consideration as potential therapeutic strategies for pancreatitis.

## Introduction

The human pancreas secretes approximately 2 liters per day of a highly alkaline fluid that transports digestive enzymes to the intestine. Acinar cells synthesize and secrete these digestive enzymes, which are initially released into isotonic fluid with a near-neutral pH. Pancreatic ductal cells are responsible for secreting the majority of the fluid of pancreatic juice (Lee et al., 2012, 2021). A key function of the ductal epithelium is the absorption of chloride (Cl^-^) ions and their exchange for bicarbonate (HCO_3_^-^), a process that increases the HCO_3_^-^ concentration of pancreatic juice to levels exceeding 140 mM (Shin et al., 2020). HCO_3_^-^ is the principal biological buffer and plays essential roles in pH regulation across all tissues. In the pancreas, however, HCO_3_^-^ serves additional specialized functions, including neutralization of gastric acid, prevention of premature activation of digestive enzymes during their transit to the intestine, and establishment of an optimal intestinal pH for digestive enzyme activity (Lee et al., 2012). Moreover, as a chaotropic molecule, HCO_3_^-^ facilitates the secretion and solubilization of macromolecules such as digestive enzymes and mucins in biological fluids (Hatefi and Hanstein, 1969; Quinton, 2010).

Pancreatic enzyme, fluid, and HCO_3_^-^ secretion are highly regulated processes that are initiated in response to food intake. The digestive response begins with neurohumoral stimulation of enzyme secretion from acinar cells. Activation of Ca²^+^-mobilizing Gq-coupled receptors (GPCRs), most notably muscarinic type 3 receptors (M3R), leads to an increase in cytoplasmic free Ca²^+^ concentration ([Ca²^+^]_i_) (Petersen and Tepikin, 2008; Ahuja et al., 2020). The Ca²^+^-dependent signaling is synergistically potentiated by cyclic adenosine monophosphate (cAMP) signaling, amplifying the secretory response (Williams, 2019; Ahuja et al., 2020). In parallel, ductal fluid and HCO_3_^-^ secretion is predominantly stimulated by the hormone secretin acting through Gs-coupled receptors that elevate intracellular cAMP level (Lee et al., 2012; Pallagi et al., 2015). Ca²^+^ signaling downstream of M3 and other Gq-coupled receptors further synergizes with the cAMP-dependent pathway to generate a coordinated physiological ductal secretory response (Park et al., 2013; Hong et al., 2014; Luscher et al., 2020). Beyond these primary regulatory mechanisms, pancreatic secretion is finely tuned by additional modulatory factors released under various physiological conditions, including insulin, somatostatin, purines, and prostaglandins (Gardner and Jensen, 1986; Chandra, 2015).

The essential role of pancreatic HCO_3_^-^ secretion in maintaining pancreatic health is highlighted by cystic fibrosis, in which pancreatic damage is among the earliest manifestations of the disease (Durie, 2000). In addition, impaired HCO_3_^-^ secretion has been reported in multiple forms of pancreatitis (Balazs and Hegyi, 2015; Pallagi et al., 2015; Zeng et al., 2017). To better understand the contribution of pancreatic ducts and CFTR to the initiation and progression of acute and chronic pancreatitis, it is therefore critical to elucidate the molecular mechanisms that mediates ductal HCO_3_^-^ secretion. Comprehensive discussions of pancreatic fluid and HCO_3_^-^ secretion and their regulation are available in a recent review (Lee et al., 2021), along with molecular insights into the role of STIM1-mediated ER/PM junctions in the regulation of anoctamin 1 (ANO1) (Lin et al., 2025) and CFTR (Sarkar et al., 2025). Here, we summarize the key features of ductal fluid and HCO_3_^-^ secretion.

## Ductal fluid and HCO_3_^-^ secretion

**Figure 1:**
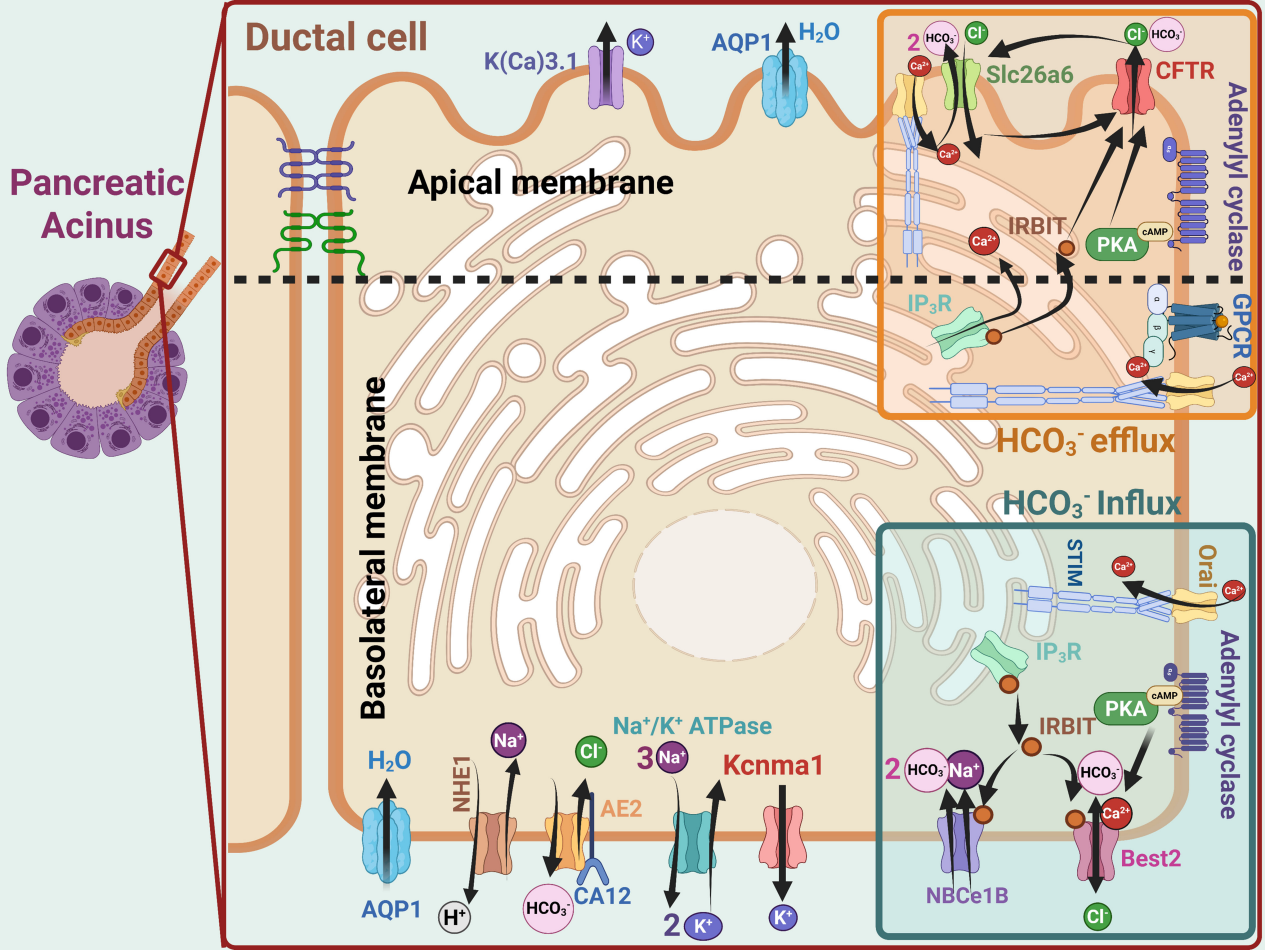
A model of ductal fluid and HCO_3_^-^ secretion. The model summarizes the principal basolateral (highlighted in lower right) and luminal (highlighted in upper right) transporters that mediate ductal fluid and HCO_3_^-^ secretion. HCO_3_^-^ enters epithelial cells primarily through the basolateral, electrogenic 1Na^+^–2HCO_3_^-^ cotransporter NBCe1-B, with additional influx mediated by the electroneutral Na^+^/H^+^ exchanger NHE1. The Cl^-^/HCO_3_^-^ channel Best2 likely also contribute to basolateral HCO_3_^-^ uptake and facilitate clearance of cytoplasmic Cl^-^. HCO_3_^-^ subsequently exits across the luminal membrane mainly via the CFTR-regulated, electrogenic 1Cl^-^/2HCO_3_^-^ exchanger SLC26A6, while CFTR primarily functions to recycle Cl^-^. Under conditions of low luminal and intracellular Cl^-^ concentrations, residual HCO_3_^-^ is secreted directly through CFTR following an increase in its HCO_3_^-^ permeability. Most luminal Cl^-^ is reabsorbed and is likely extruded across the basolateral membrane through Best2. The electrogenic basolateral and luminal HCO_3_^-^ transporters NBCe1-B and SLC26A6, respectively, mediate net osmolyte transport which, together with paracellular Na^+^ flow from the basolateral to the luminal side, drives transepithelial water secretion.

Pancreatic fluid and HCO_3_^-^ secretion is a tightly coupled, vectorial process mediated by the coordinated activity of basolateral and luminal HCO_3_^-^ transporters. Over the past two decades, molecular, cellular, and physiological studies have identified the key ductal transporters and elucidated their properties and regulatory mechanisms, as summarized in **Figure 1**. The energy driving ductal secretion is provided by the Na^+^ and K^+^ gradients generated by the basolateral 3Na^+^/2K^+^-ATPase. The Na^+^ gradient fuels Na^+^-coupled transporters, whereas K^+^ channels establish a negative membrane potential that supports electrogenic transporters. HCO_3_^-^ secretion is initiated by basolateral HCO_3_^-^ uptake, which occurs predominantly via the electrogenic 1Na^+^–2HCO_3_^-^ cotransporter NBCe1-B (SLC4A4) (Zhao et al., 1994; Abuladze et al., 1998; Boron et al., 2009). In addition, basolateral pH homeostasis is maintained by the Na^+^/H^+^ exchanger NHE1 (SLC9A1) (Lee et al., 2000) and the Cl^-^/HCO_3_^-^ exchanger AE2 (SLC4A2) (Roussa et al., 2001). By extruding cytoplasmic H^+^, NHE1 contributes approximately 25% of basolateral HCO_3_^-^ influx (Lee et al., 2012), while AE2 plays a critical role in maintaining intracellular Cl^-^ required to sustain HCO_3_^-^ secretion. Another basolateral Cl^-^ and HCO_3_^-^ transporter is the Ca²^+^-activated channel Bestrophin 2 (Best2) (Qu and Hartzell, 2008; Yu et al., 2010; Zhang et al., 2010), which is permeable to both anions (Qu and Hartzell, 2008). In the colon, Best2 mediates HCO_3_^-^ influx (Yu et al., 2010); however, its function in pancreatic ducts remains unclear. Best2 may correspond to the basolateral Ca²^+^-activated Cl^-^ channel proposed to mediate the “push–pull” model of Cl^-^ transport in pancreatic acinar and duct cells (Kasai and Augustine, 1990).

Basolateral HCO_3_^-^ influx is followed by HCO_3_^-^ efflux across the apical membrane, which is mediated by coordinated physical and functional interactions between the cAMP-activated Cl^-^ channel CFTR (Csanady et al., 2019) and the electrogenic 1Cl^-^/2HCO_3_^-^ exchanger SLC26A6 (Shcheynikov et al., 2006; Wang et al., 2006). The channel function of CFTR mediates Cl^-^ and subsequently HCO_3_^-^ flow (Lee et al., 1999), with SLC26A6 initially serving as the primary exchanger responsible for pancreatic luminal HCO_3_^-^ secretion (Wang et al., 2006; Stewart et al., 2009). CFTR and SLC26A6 are mutually regulated through direct protein–protein interactions. Specifically, the CFTR regulatory domain (RD) interacts with the sulfate transporter and anti-sigma factor antagonist (STAS) domain of SLC26A6, resulting in obligatory reciprocal activation of both transporters (Ko et al., 2004). This regulatory mechanism is conserved across the SLC26A family (Geertsma and Oliver, 2024), including SLC26A3, which is expressed in the pancreatic duct (Stewart et al., 2011). In addition to SLC26A6, CFTR also regulates the Cl^-^ channel SLC26A9 (Dorwart et al., 2007; Geertsma and Oliver, 2024). Genetic linkage analyses indicate that SLC26A9 exhibits the strongest association with cystic fibrosis severity among CFTR modifier genes (Sun et al., 2012). Moreover, SLC26A9 variants are associated with pancreatic damage in cystic fibrosis (Sun et al., 2012) and with pancreatitis (Balazs and Mall, 2018). However, the precise role of SLC26A9 in pancreatic ductal secretion remains unclear. Although electrogenic SLC26A6 mediates the bulk of luminal HCO_3_^-^ secretion, its activity becomes self-limiting as luminal HCO_3_^-^ concentrations exceed 100 mM and intracellular Cl^-^ levels decline to approximately 7 mM (Ishiguro et al., 2002) during active secretion. Under these conditions, SLC26A6-mediated exchange is inhibited before reaching the ~140 mM HCO_3_^-^ concentration observed in pancreatic juice. This shift promotes increased HCO_3_^-^ permeability of CFTR (Park et al., 2010), allowing CFTR to complete the HCO_3_^-^ secretory process. CFTR HCO_3_^-^ permeability is further modulated by the Cl^-^-sensitive protein kinase With-No-Lysine Kinase 1 (WNK1) (Park et al., 2010; Kim et al., 2020).

Electrogenic HCO_3_^-^ influx via NBCe1-B and apical HCO_3_^-^ efflux via SLC26A6 generate net electrolyte secretion, which, together with serosal-to-luminal Na^+^ flow across the tight junctions, establishes an osmotic gradient driving fluid secretion by the pancreatic duct. Water movement is facilitated by aquaporin channels. Aquaporin 1 (AQP1) is expressed on both basolateral and luminal membranes in the mouse pancreatic duct (Furuya et al., 2002; Burghardt et al., 2003; Venglovecz et al., 2018), but is restricted to the luminal membrane in the human pancreatic duct (Venglovecz et al., 2018). Consistent with its functional importance, deletion of AQP1 in mice nearly abolishes pancreatic ductal HCO_3_^-^ and fluid secretion (Venglovecz et al., 2018).

## CFTR

The importance of HCO_3_^-^ secretion, and consequently of CFTR, in pancreatic function became evident with the initial description of cystic fibrosis (CF), which was originally diagnosed as cystic fibrosis of the pancreas (Quinton, 1999). Early studies further demonstrated that pancreatic juice is highly alkaline under physiological conditions, whereas it is abnormally acidic in patients with CF (Johansen et al., 1968). The identification of CFTR as the protein mutated in CF (Kerem et al., 1989; Riordan et al., 1989; Rommens et al., 1989) enabled detailed investigation of its role in pancreatic ductal secretion (Lee et al., 2012). CFTR is localized to the apical membrane of pancreatic duct cells, as well as other secretory epithelia, and is activated by the cAMP/PKA signaling pathway (Marino et al., 1991; Zeng et al., 1997). The highest and most dominant expression of CFTR in the duct is in ionocytes (Shah et al., 2022; Uchida et al., 2026). The function of ionocytes has been examined in the salivary gland duct (Shah et al., 2022; Uchida et al., 2026); however, the presence and function of ionocytes in the pancreatic duct have not yet been demonstrated. The levels of ionocytes and CFTR are reduced in Sjögren’s disease, and the reduction in CFTR appears to correlate with disease severity (Zhang et al., 2022). Targeted deletion of ionocytes in salivary glands results in more acidic saliva (Uchida et al., 2026), highlighting the role of ductal ionocytes in HCO_3_^-^ secretion, which may also be altered in pancreatitis. CFTR exhibits context-dependent ion selectivity that is critical for pancreatic duct function. When cytoplasmic and luminal Cl^-^ concentrations exceed ~10–20 mM, CFTR functions predominantly as a Cl^-^ channel, supporting the activity of SLC26A6 and promoting Cl^-^ absorption from pancreatic juice (**Figure 1** and (Linsdell et al., 1997; Shcheynikov et al., 2004; Lee et al., 2008)). By contrast, when either intracellular (Park et al., 2010; Jun et al., 2016) or luminal Cl^-^ concentrations (Shcheynikov et al., 2004) fall below ~10 mM, CFTR pore selectivity shifts toward HCO_3_^-^, resulting in increased HCO_3_^-^/Cl^-^ selectivity ratio and net luminal HCO_3_^-^ efflux (Ishiguro et al., 2009). This Cl^-^-dependent change in CFTR selectivity is mediated by the Cl^-^-sensitive kinase WNK1 (Piala et al., 2014; Kim et al., 2020). Reduced intracellular Cl_-in_ also activates WNK4, which is more sensitive to Cl_-in_ than WNK1 (Terker et al., 2016). WNK4 inhibits CFTR and other HCO_3_^-^, Na^+^, Cl^-^, and K^+^ transporters (Luscher et al., 2020), thereby fine-tuning epithelial transport processes and physiological function (Yang et al., 2011; Piala et al., 2014; Terker et al., 2016). Notably, regulation of CFTR by WNK1 appears particularly relevant in CFTR mutants associated with chronic pancreatitis in the absence of classic CF symptoms (Larusch et al., 2014; Kim et al., 2020).

CFTR activation by cAMP signaling involves the generation of cAMP by adenylyl cyclases (ACs), leading to PKA-mediated phosphorylation of the CFTR regulatory domain (Csanady et al., 2019). This signaling pathway is spatially organized by A-kinase anchoring proteins (AKAPs), which assemble cAMP signaling components into discrete cellular microdomains (Omar and Scott, 2020). Although the specific AKAPs and AC isoforms that regulate CFTR in pancreatic ducts remain unknown, soluble AC10 (Wang et al., 2005) and Ca²^+^-activated AC1 (Namkung et al., 2010) have been implicated in CFTR activation in airway epithelia, whereas AC6 plays a dominant role in the intestine (Thomas et al., 2018). Importantly, under physiological conditions, CFTR activity is not determined by cAMP alone but instead reflects synergistic integration of cAMP and Ca²^+^ signaling pathways (Ahuja et al., 2014). This synergy is mediated by the regulatory protein IRBIT (IP_3_ receptor-binding protein released with IP_3_) (Yang et al., 2009). In resting cells, IRBIT binds to IP_3_ receptors (IP_3_Rs), competing with IP_3_ and suppressing Ca²^+^ release. Upon physiological stimulation of IP_3_-generating GPCRs, modest increases in IP_3_ promote its binding to IP_3_Rs, leading to dissociation of IRBIT. Freed IRBIT subsequently interacts with downstream targets, including CFTR (Yang et al., 2009) and the Na^+^-HCO_3_^-^ cotransporter NBCe1-B (Shirakabe et al., 2006; Yang et al., 2009), thereby facilitating their activation. Both cAMP and Ca²^+^ signaling pathways are highly compartmentalized at membrane contact sites (MCS), particularly at endoplasmic reticulum–plasma membrane (ER/PM) junctions, where they coordinate transporters regulation (Ahuja et al., 2014; Muallem et al., 2017). These ER/PM junctions are assembled by tethering proteins, many of which function as lipid transfer proteins, including extended synaptotagmins (E-Syts) (Saheki and De Camilli, 2017) and oxysterol-binding protein–related proteins (ORPs) (Arora et al., 2022). At these sites, signaling pathways and transporters are regulated by membrane lipids, notably phosphatidylserine (PtdSer), PI(4)P, and PI(4,5)P_2_ (Chung et al., 2023; Lin et al., 2025; Sarkar et al., 2025). In pancreatic (and salivary) ducts, E-Syt3 controls PtdSer abundance at ER/PM junctions, thereby regulating CFTR and NBCe1-B activity and ultimately determining ductal fluid and HCO_3_^-^ secretion (Sarkar et al., 2025).

## CFTR, HCO_3_^-^ secretion, and pancreatitis

The close relationship between CFTR dysfunction and pancreatitis is evident from studies of patients with cystic fibrosis (CF). As noted above, one of the earliest clinical manifestations of CF is pancreatic insufficiency (Durie, 2000). Importantly, even CF patients who retain sufficient residual exocrine function and are classified as pancreatic-sufficient remain at increased risk of developing pancreatitis (Ooi and Durie, 2012). The first direct link between CFTR and pancreatitis was established through the identification of a strong association between CFTR gene mutations, including the 5T variant, and chronic pancreatitis (Cohn et al., 1998; Sharer et al., 1998). Subsequent studies demonstrated robust genotype–phenotype correlations between specific CFTR mutations and multiple forms of pancreatitis (Dray et al., 2003; Ooi and Durie, 2012). In addition, CFTR mutations further increase the risk of pancreatitis in individuals carrying mutations in the trypsin inhibitor gene PSTI (SPINK1) (Cohn, 2005; Schneider et al., 2011). CFTR dysfunction has also been implicated in disease severity, as patients with CFTR mutations associated with autoimmune pancreatitis show a delayed response to corticosteroid therapy (Chang et al., 2015).

The contribution of impaired HCO_3_^-^ secretion to pancreatitis has been recognized since the 1960s and 1970s (Zoppi et al., 1970; Kaiser and Drack, 1974). However, following the discovery of CFTR as a Cl^-^ channel essential for epithelial fluid and electrolyte secretion, research in CF largely focused on defective Cl^-^ transport as the primary pathogenic mechanism. This emphasis led, at least in part, to underappreciation of the role of HCO_3_^-^ transport and of CFTR as a regulator of epithelial HCO_3_^-^ secretion (Quinton, 2001). Renewed attention to HCO_3_^-^ transport emerged with the first report of CFTR mutations that preferentially impair HCO_3_^-^ permeability while relatively sparing Cl^-^ conductance (Choi et al., 2001). More recent and particularly informative studies have identified additional CFTR mutations in patients with pancreatitis who do not exhibit classical symptoms of CF (Weiss et al., 2009; Larusch et al., 2014). These variants preserve normal or near-normal Cl^-^ channel activity but show markedly reduced HCO_3_^-^ permeability and transport (Choi et al., 2001; Jun et al., 2016; Kim et al., 2020). Meta-analyses further demonstrate that carriers of these mutations have a significantly increased risk of developing chronic pancreatitis (Berke et al., 2022).

**Figure 2:**
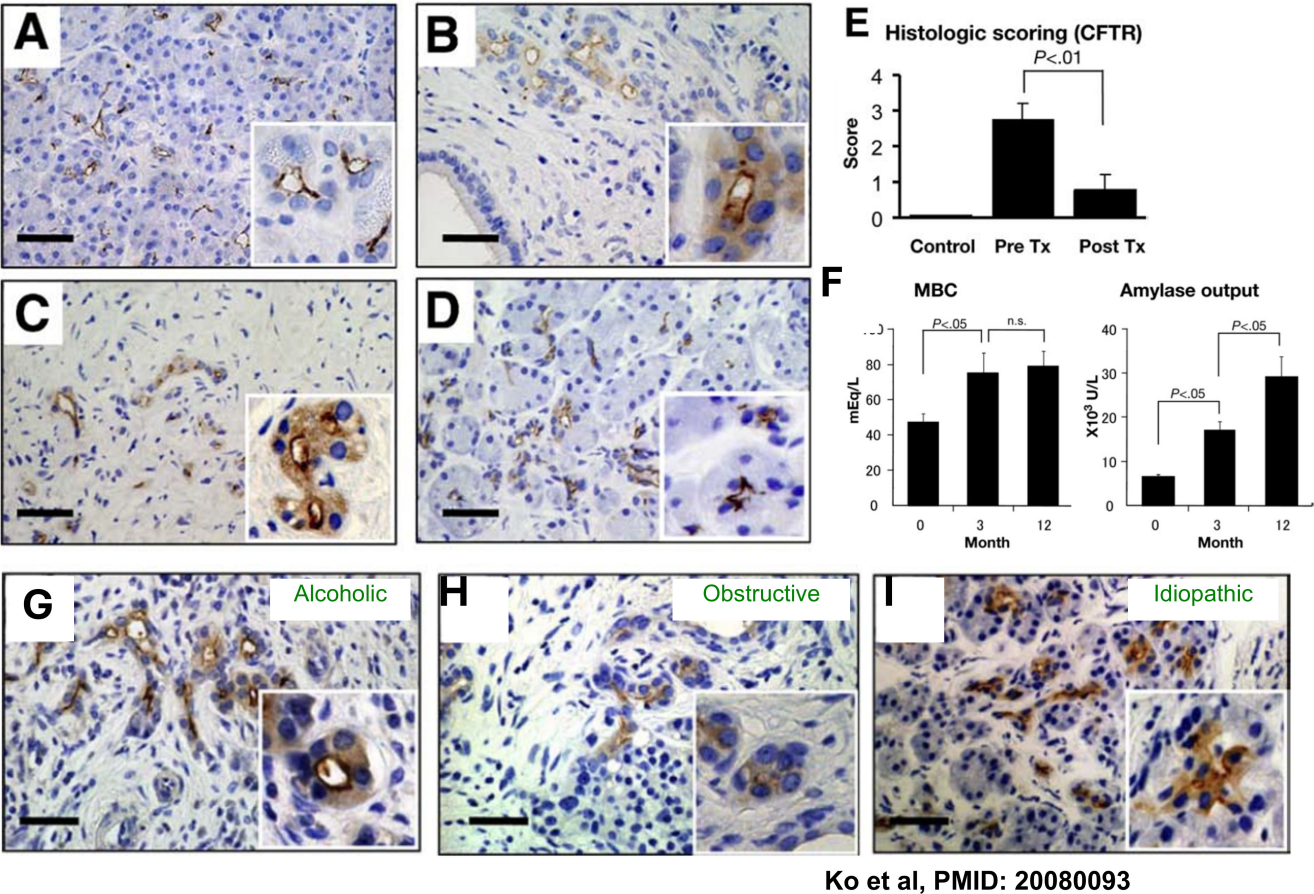
Immunolocalization of CFTR in the pancreas and pancreatic exocrine function. Immunolocalization of CFTR in the pancreas. **(A)** Normal subject. **(B)** AIP, surgically resected. **(C, D)** Biopsy specimens from the same patient **(C)** before and **(D)** after corticosteroid treatment. **(E)** Pancreatic sections obtained from normal subjects (n = 4) and from AIP patients before treatment (0 months; n = 7) or after 3 months of corticosteroid therapy (n = 7) were scored for the degree of cytoplasmic CFTR staining in pancreatic ducts as 0 (none), 1 (slight), 2 (moderate), or 3 (severe). **(F)** Changes in pancreatic exocrine function at 0, 3, and 12 months after initiation of corticosteroid therapy (n = 3), shown as (left) maximal HCO_3_^-^ concentration (MBC) and (right) amylase output. CFTR localization was also examined in pancreatic tissue from patients with **(G)** alcoholic, **(H)** obstructive, and **(I)** idiopathic chronic pancreatitis. Each panel represents 6 alcoholic, 2 obstructive, and 3 idiopathic pancreatitis cases, respectively. Scale bars, 20 μm. Insets show higher-magnification images. Data are reproduced from Ko et al (Ko et al., 2010).

The recognition that CFTR plays a major role in pancreatitis led to a landmark study examining the relationship between CFTR expression and pancreatic disease in humans (Ko et al., 2010). The study first demonstrated that CFTR is mislocalized from the luminal membrane in patients with autoimmune, idiopathic, obstructive, and alcoholic pancreatitis (**Figures 2A–D, G–I**). In addition, pancreatic biopsies from patients with autoimmune pancreatitis obtained before and after corticosteroid treatment showed that steroid therapy restored normal CFTR localization (**Figures 2D, E**). To assess the functional consequences of CFTR dysfunction, the authors measured ductal fluid and HCO_3_^-^ secretion as well as acinar cells amylase secretion. Remarkably, corticosteroid treatment increased both HCO_3_^-^ and amylase secretion (**Figure 2F**), demonstrating a critical role for CFTR in ductal secretion and underscoring the close functional interrelationship between ductal and acinar compartments. These findings indicate that impaired ductal function is a common feature of multiple forms of pancreatitis and, importantly, that restoration of ductal CFTR activity is sufficient to rescue acinar cell function (Ko et al., 2010). The correction of CFTR by corticosteroid treatment suggests that CFTR degradation is the result of upstream inflammation and inflammatory mediators Consistent with these findings, subsequent studies further implicated CFTR dysfunction in pancreatitis. In alcoholic patients with pancreatitis, CFTR function, assessed by sweat Cl^-^ measurements, was impaired, and CFTR protein levels were reduced in pancreatic ducts (Maleth et al., 2015). Similarly, assessment of CFTR activity using nasal potential difference revealed defective CFTR function in patients with chronic pancreatitis (Schlosser et al., 2022). More recently, pancreatic organoids generated from patients with idiopathic, hereditary, and alcohol-related pancreatitis demonstrated CFTR dysfunction across all disease etiologies (Osorio-Vasquez et al., 2025). This organoid-based model provides a powerful platform for mechanistic studies and holds significant promise for both diagnostic applications and therapeutic development (see below).

The human findings were corroborated in guinea pig and mouse models of pancreatitis. Exposure to alcohol inhibited CFTR function in epithelial cell lines as well as in pancreatic duct cells isolated from mice and guinea pigs (Judak et al., 2014; Maleth et al., 2015). Similar inhibition of CFTR activity was observed following treatment with the non-oxidative alcohol metabolites palmitoleic acid ethyl ester and palmitoleic acid (Judak et al., 2014). Mechanistically, ethanol impaired ductal fluid and HCO_3_^-^ secretion through multiple effects on CFTR. Alcohol treatment reduced CFTR mRNA expression, decreased CFTR protein stability, disrupted proper folding in the endoplasmic reticulum, and impaired trafficking of CFTR to the cell surface (Maleth et al., 2015).

## Correcting CFTR as a promising treatment for pancreatitis

**Figure 3:**
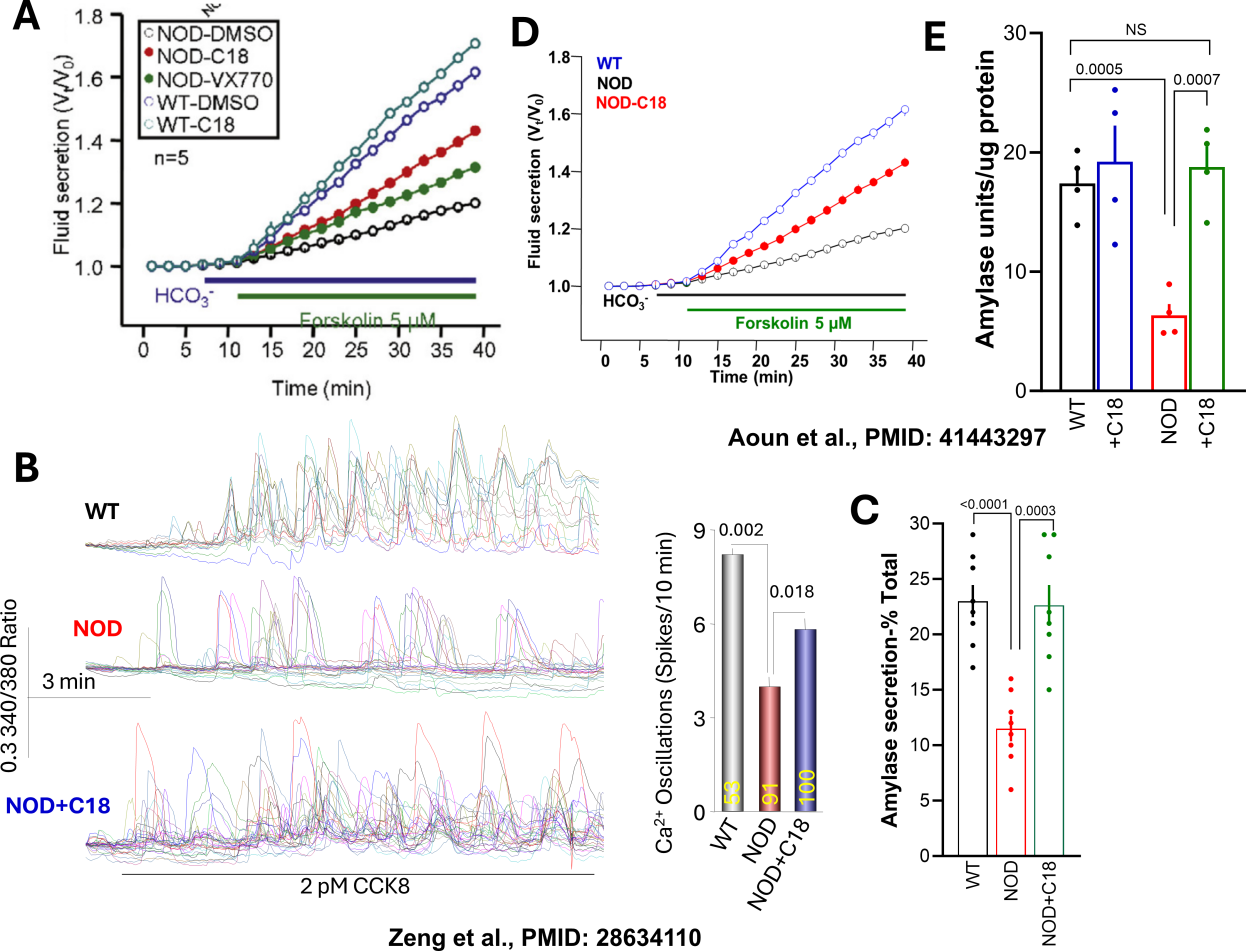
Pancreatic and salivary glands ductal fluid secretion and acinar amylase exocytosis in NOD mice before and after treatment with CFTR modulators. Ductal fluid secretion was measured in microdissected intralobular ducts from the pancreas **(A)** or parotid gland **(D)**, and amylase secretion was measured in acinar clusters isolated from the pancreas **(C)** or parotid gland **(E)**. Ca²^+^ oscillations triggered by 2 pM CCK8 were measured in pancreatic acinar cells **(B)**. Cells were obtained from wild-type or NOD (Non-Obese Diabetic) mice treated with DMSO (vehicle), the CFTR corrector C18, or the CFTR potentiator VX-770, as indicated in the Figure. NOD mice develop a spontaneous, progressive inflammatory disease that leads to the destruction of pancreatic islets between 13 and 17 weeks of age. The mice were used at 11–12 weeks, when significant inflammation is present in the pancreas and salivary glands but before the onset of diabetes. Pancreatic data are reproduced from Zeng et al (Zeng et al., 2017), and parotid gland data are reproduced from Aoun et al (Aoun et al., 2026).

The observation of mislocalized CFTR in pancreatitis patients using multiple experimental approaches (Ko et al., 2010) suggested that restoration of proper CFTR localization and function could represent a promising therapeutic strategy. Although corticosteroids are commonly used in some forms of pancreatitis, their clinical utility is limited by significant side effects. In contrast, the development of CFTR modulators, including correctors that improve CFTR folding, expression, and apical localization, and potentiators that enhance channel gating, has yielded drugs with excellent efficacy and tolerability (Tummler et al., 2025; Wang et al., 2025). These advances raise the possibility of repurposing CFTR modulators for the treatment of pancreatitis and other exocrine gland disorders. Preclinical studies in animal models of pancreatitis provide strong support for this approach. CFTR protein expression and function are reduced in several models of chronic pancreatitis, autoimmune pancreatitis, and Sjögren’s disease (Zeng et al., 2017). As illustrated in **Figure 3**, treatment of mouse models with a single CFTR corrector (C18, similar to VX-809) was sufficient to restore CFTR expression and ductal fluid secretion in both pancreatic and salivary gland ducts (**Figures 3A, D**). Importantly, correction of CFTR dysfunction also rescued acinar cell function, normalizing Ca²^+^ signaling and amylase secretion (**Figures 3B, C, E** (Zeng et al., 2017)). It is of note that treatment with C18 was after establishment of the disease, as would be the case in treating patients with pancreatitis. The findings with the CFTR corrector were further validated in studies using combinations of CFTR correctors and potentiators in models of acute pancreatitis. In one study, administration of a CFTR corrector (VX-661) together with a potentiator (VX-770) prior to induction of acute pancreatitis preserved CFTR expression and ductal function and significantly reduced pancreatic injury in mice (Fur et al., 2021). Similarly, pretreatment of guinea pigs with alcohol-induced acute pancreatitis using the CFTR potentiator ivacaftor (VX-770) and the corrector lumacaftor (VX-809) preserved apical CFTR expression and activity in ductal cells and attenuated pancreatic damage (Venglovecz et al., 2024). Collectively, these findings highlight the broad therapeutic potential of CFTR modulators for the treatment of multiple forms of pancreatitis.

The most effective current therapy for cystic fibrosis (CF) is a triple combination regimen consisting of two CFTR correctors, tezacaftor and elexacaftor, and the CFTR potentiator ivacaftor (Tummler et al., 2025; Wang et al., 2025). Multiple studies have reported improved pancreatic function and reduced symptoms of chronic pancreatitis in patients treated with CFTR modulators. For example, ivacaftor treatment restored pancreatic sufficiency in a pancreatic-insufficient CF patient (Megalaa et al., 2019), and treatment of 3 CF patients carrying combined CFTR mutations (ΔF508/G551D or ΔF508/3272-26A>G) resulted in recovery of exocrine pancreatic function (Munce et al., 2020). In addition, a year-long study of 12 patients homozygous for the ΔF508 mutation treated with lumacaftor and ivacaftor demonstrated improved pancreatic function (Yaacoby-Bianu et al., 2022). Importantly, a prospective study reported a reduced incidence of recurrent acute pancreatitis episodes in 15 CF patients with pancreatic sufficiency treated with ivacaftor alone or in combination with other CFTR modulators (Akshintala et al., 2019). These clinical findings are further supported by a study using pancreatic organoids derived from 36 patients with idiopathic, hereditary, and alcohol-related chronic pancreatitis, which showed reduced CFTR expression and function that were restored by CFTR modulator treatment, accompanied by decreased mitogenic and inflammatory signaling (Osorio-Vasquez et al., 2025). Together, these data strongly support a pathogenic role for CFTR dysfunction in pancreatitis and demonstrate its therapeutic reversibility.

Despite these promising studies, several challenges remain regarding the use of CFTR correctors. First, the high cost of treatment-largely driven by the relatively small population of patients with cystic fibrosis-limits its accessibility. Costs may decrease if these therapies are extended to larger patient populations, such as those with pancreatitis. Another limitation is that CFTR correctors and potentiators may not restore CFTR expression and function rapidly enough to be effective in acute pancreatitis. Moreover, additional studies are needed to further establish the efficacy of CFTR-targeting drugs in correcting CFTR dysfunction in the human pancreas and to address existing gaps in the evidence. Notably, there is currently a lack of well-designed, controlled clinical trials with sufficiently large patient cohorts to thoroughly evaluate the use of CFTR correctors and potentiators in the treatment of pancreatitis.

Several important conclusions emerge from the combined evidence obtained from animal models and studies in patients. First, CFTR expression and function are compromised across all forms of acute and chronic pancreatitis. Second, ductal fluid and HCO_3_^-^ secretion are essential for maintaining pancreatic integrity and homeostasis. Third, the improvement of pancreatic ductal function and sufficiency observed in patients with CF and pancreatitis treated with CFTR modulators indicates recovery of acinar cell function secondary to restoration of ductal secretion. Accordingly, both human and animal studies support the concept that targeting ductal dysfunction promotes repair of acinar tissue and restores pancreatic function. These findings suggest that CFTR modulators should be carefully evaluated as therapeutic agents for pancreatitis, either alone or in combination with other treatment modalities, such as Orai1 inhibitors (Pallagi et al., 2020) or calcineurin inhibitors (Barakat et al., 2022).
